# Inflammation and nutrition based screening tests for detection of infection in cases of rapid hip destruction

**DOI:** 10.1038/s41598-022-07678-3

**Published:** 2022-03-04

**Authors:** Koki Abe, Hyonmin Choe, Masatoshi Oba, Taro Tezuka, Hiroyuki Ike, Naomi Kobayashi, Yutaka Inaba

**Affiliations:** 1 Department of Orthopaedic Surgery, Yokohama City University, 3-9 Fukuura, Kanazawa-Ku, Yokohama, 236-0004 Japan; 2 Department of Orthopaedic Surgery, Yokohama City University Medical Center, Yokohama, Japan

**Keywords:** Medical research, Biomarkers

## Abstract

Preoperative diagnosis of infection is important for appropriate surgical treatment of patients with rapid hip destruction (RHD). We investigated whether test results, including inflammatory and nutritional markers, could be used to accurately differentiate infectious and non-infectious RHD. Fifty patients with RHD who underwent total hip arthroplasty within a year of onset were observed. Infectious RHD was defined as ≥ 2 positive serological inflammatory, microbiological, or pathological evaluations. The albumin to globulin ratio (AGR), C-reactive protein (CRP)/albumin ratio (CAR), Glasgow prognostic score (GPS), modified GPS (mGPS), prognostic nutritional index (PNI), geriatric nutritional risk index (GNRI), and platelet to lymphocyte ratio (PLR) were calculated from the blood test results. In the infectious group, the white blood cell count, platelet count, CRP level, erythrocyte sedimentation rate, CAR, GPS, mGPS, and PLR were significantly higher, while the albumin level, AGR, PNI, and GNRI were significantly lower. The CRP and albumin levels showed the highest sensitivity (1.00 for both; specificity of 0.87 and 0.73, respectively) in diagnosing infectious RHD. Combining these measurements (CAR) increased the specificity to 0.92. The accuracy of other nutritional assessments was good. Thus, nutritional assessment as well as conventional assessment of the inflammatory response can improve the accuracy of preoperative diagnosis of infectious RHD.

## Introduction

Rapidly destructive coxarthrosis (RDC) is an emergent disorder characterized by the progressive destruction of the hip joint due to unknown causes^1–4^. It may be associated with subchondral insufficiency fracture, leading to rapid hip joint destruction within a year and has no apparent cause. However, infections, osteonecrosis, inflammatory diseases, and Charcot arthropathy may also show radiographic findings (rapid hip destruction [RHD]) similar to RDC^5^. Therefore, RHD was defined as a condition of accelerated hip deterioration within one year. RHD causes severe pain and inactivity in patients, and total hip arthroplasty (THA) is one of the most optimal surgical treatments. In non-infectious RHD patients, THA can effectively relieve pain, restore hip function, and improve the quality of life^6^, although performing THA in infectious RHD may generate the devastating result of periprosthetic joint infection (PJI). Careful preoperative diagnosis is necessary for the selection of the surgical treatment for infectious RHD patients, i.e. two-staged THA^7,8^.Therefore, surgical treatment should be based on the diagnosis of infectious or non-infectious RHD.

Preoperative infection diagnosis is often difficult in RHD patients, because RHD is an amalgam of conditions that cause destructive changes in the hip and lacks classification based on underlying diseases. Several hip disorders may cause RHD, including non-infectious diseases of subchondral fractures, osteonecrosis of the femoral head (ONFH), rheumatoid arthritis, musculoskeletal tumor, and the infectious disease of septic arthritis of the hip (SAH)^4,5^. Imaging findings using X-rays play only a small role in the differentiation, but magnetic resonance imaging (MRI) may produce effective findings. However, only a few studies have focused on infectious RHD patients’ bone and soft tissue abnormalities^9,10^.

Detection of causative bacteria in a preoperative joint aspiration culture (PAC) is the most specific option for the early differential diagnosis of infectious RHD, but a microbiological culture of synovial fluid is not sufficiently sensitive for infection diagnosis, due to the possibility of false negative results or a dry tap^11,12^. Serological assessment of inflammatory markers is a sensitive diagnostic option for infection screening, but clinical usefulness is limited due to lack of specificity within the current diagnostic application of inflammatory markers^13,14^. Previously, studies demonstrated the importance of nutritional serum markers for the evaluation of systemic immunity and inflammation in proximal femur fractures, periprosthetic joint infections, surgical site infections, and tumor-related areas^15–19^. Thus, the accuracy of preoperative infection diagnosis in RHD patients can be improved by the examination of preoperative nutritional biomarkers; this has not been investigated previously.

The purpose of this retrospective study was to investigate the difference in X-ray or MRI findings and serum inflammatory and nutritional markers between infectious and non-infectious RHD and to determine whether the preoperative diagnostic accuracy of infectious RHD could be improved through these assessments.

## Results

**Figure 1:**
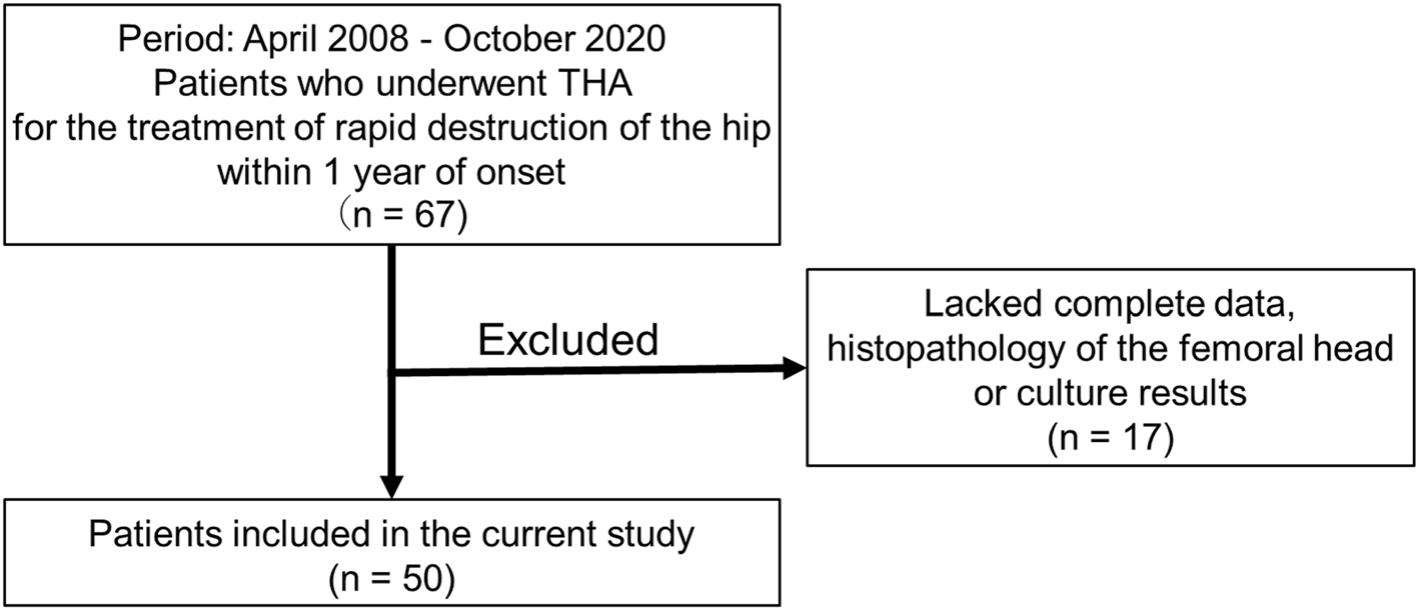
Study flowchart of all cases.

**Figure 2:**
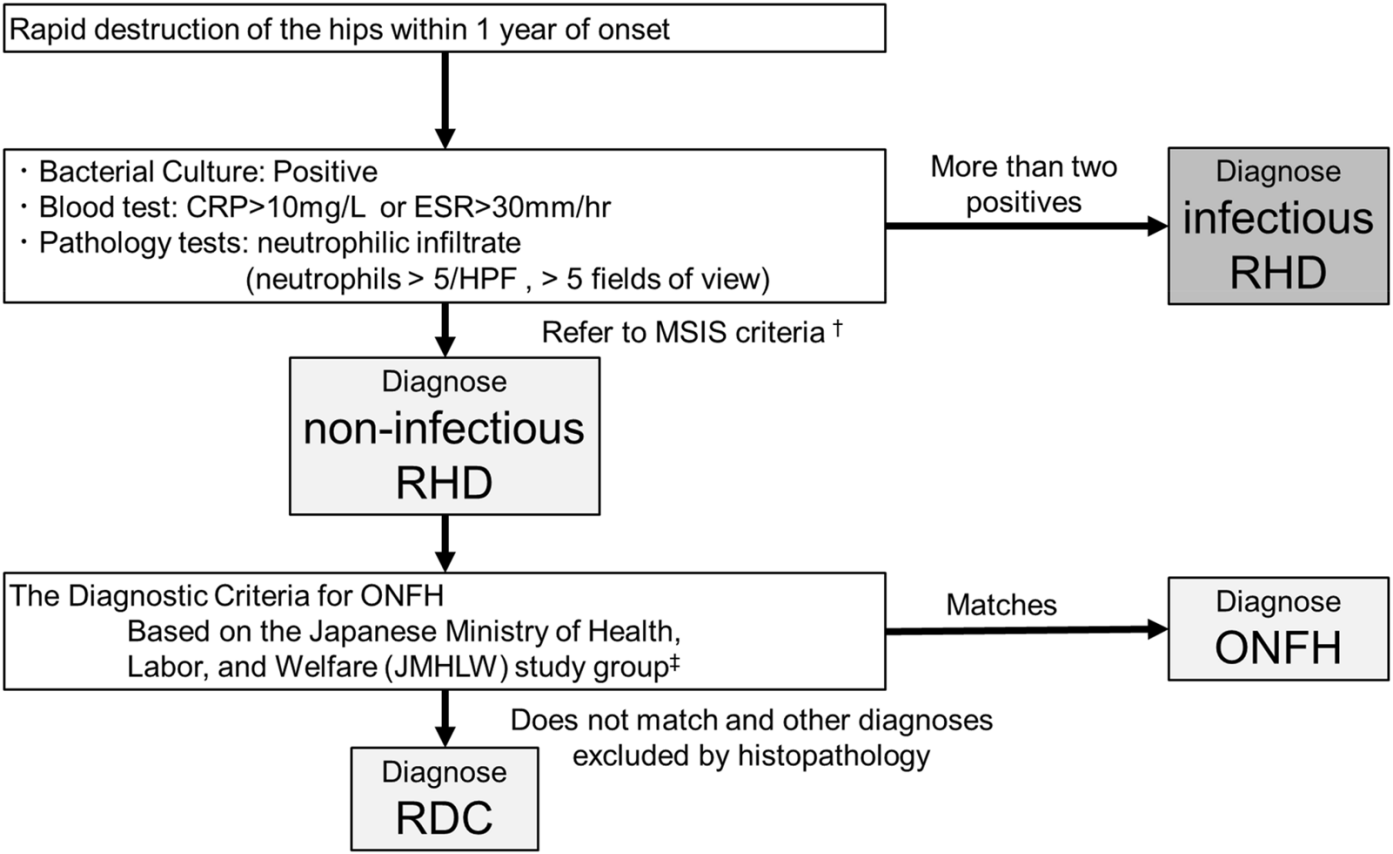
Diagnostic flowchart. *CRP* C-reactive protein, *ESR* Erythrocyte sedimentation rate, *HPF* High-powered field, *MSIS* Musculoskeletal Infection Society, *ONFH* Osteonecrosis of the femoral head, *RDC* Rapidly destructive coxarthrosis, *RHD* Rapid hip destruction. ^†^Ref. 32; ^‡^Ref. 38.

Among the 50 eligible patients (50 hips) with RHD, 13 patients were diagnosed with infectious RHD. Among the 37 patients with non-infectious RHD, 13 were diagnosed with ONFH and 24 with subchondral fracture (Figs. 1, 2, and Supplementary Table S1). There were no significant differences between the infectious and non-infected RHD patients in terms of age, gender, height, weight, body mass index (BMI), Harris hip score (HHS)^20^, time until femoral head destruction, time from onset to surgery, diabetes mellitus status, or hemodialysis. Although preoperative steroid use did not differ between groups, preoperative antibiotic use was more prevalent in infectious RHD patients (Table 1). Among non-infectious RHD patients, the RDC subgroup was significantly older, and had a higher percentage of women and less steroid use compared with the ONFH subgroup (Supplementary Table S1).

**Table 1:** Patient demographic data.

Demographic data	All patients (n = 50)	Patients with infectious RHD (n = 13)	Patients with non-infectious RHD (n = 37)	*p* value
Patient age, years (IQR)	65 (56–74)	61 (51–71)	65 (57–75)	0.868*
Female patients, n (%)	29 (58)	6 (46)	23 (62)	0.346^†^
BMI, kg/m^2^ (IQR)	23.0 (20.4–26.3)	23.5 (22.0–26.4)	22.8 (20.3–25.6)	0.413*
Time until femoral head destruction, months (IQR)	4 (2.5–6)	3 (2.5–6)	4 (2.75–6)	0.751*
Time from the first onset to surgery, months (IQR)	7 (3–10)	7.0 (3–9)	6.5 (3–12)	0.577*
Harris hip score, points (IQR)	43 (26–54)	32 (24–54)	44 (30–54)	0.617*
Diabetes mellitus, n (%)	23 (46)	4 (31)	20 (54)	0.202^†§^
Hemodialysis, n (%)	5 (10.0)	1 (7.7)	4 (10.8)	1.000^†^
Use of steroids, n (%)	15 (30)	3 (23)	12 (32)	0.728^†^
Use of antibiotics, n (%)	12 (24)	8 (62)	4 (11)	< 0.001^†§^

*BMI* Body mass index, *IQR* Interquartile range, *RHD* Rapid hip destruction.

*Mann–Whitney’s U test.

^†^Fisher’s exact test.

^§^Statistically significant.

### Inflammatory and nutritional markers

In inflammatory markers, white blood cell (WBC), leukocyte count, platelet counts, C-reactive protein (CRP), and erythrocyte sedimentation rate (ESR) were significantly higher in the patients with infectious RHD, than in non-infectious RHD (Table 2). In nutritional data, albumin levels were significantly lower in the patients with infectious RHD than non-infectious RHD, although there was no difference in total protein and glycated hemoglobin A1c (HbA1c) (Table 2).

**Table 2:** Patients laboratory data.

Laboratory data	All patients (n = 50)	Patients with infectious RHD (n = 13)	Patients with non-infectious RHD (n = 37)	*p* value
WBC count, 10*3/μL (IQR) Total patients	6.8 (5.9–8.1)	7.9 (6.3–10.5)	6.5 (5.7–7.5)	0.044*^§^
Steroids user No user	8.0 (6.3–10.6) 6.4 (5.9–7.5)	10.5(9.2–10.6) 7.4 (6.1–8.3)	7.4 (5.9–9.3) 6.3 (5.7–7.2)	N.S
Lymphocyte count, 10*3/μL (IQR)	1.2 (0.9–1.5)	1.3 (0.9–1.5)	1.2 (0.9–1.5)	0.868*
Neutrophil count, 10*3/μL (IQR)	4.9 (3.6–6.3)	5.9 (5.1–7.3)	4.6 (3.4–5.6)	0.032*^§^
Monocyte count, 10*2/μL (IQR)	4.9 (3.5–6.2)	5.8 (4.2–6.8)	4.5 (3.5–5.8)	0.284*
Platelet count, 10*4/μL (IQR)	25.7 (20.0–33.8)	45.7 (33.0–49.8)	21.4 (18.2–29.3)	< 0.001*^§^
CRP, mg/L (IQR)	6.6 (1.1–22.7)	56.4 (48.2–96.9)	2.5 (0.7–8.2)	< 0.001*^§^
ESR, mm/h (IQR)	19 (9–49)	76 (58–100)	13 (6–22)	< 0.001*^§^
HbA1c, % (IQR)	5.7 (5.3–6.2)	5.8 (5.3–6.5)	5.7 (5.4–6.1)	0.835*
Total protein, g/dL (IQR)	7.0 (5.1–8.3)	7.1 (5.1–8.3)	6.9 (5.7–8.2)	0.73^||^
Albumin, g/dL (IQR)	4.0 (3.5–4.2)	3.1 (2.7–3.6)	4.1 (3.9–4.3)	< 0.001*^§^

*CRP* C-reactive protein, *ESR* Erythrocyte sedimentation rate, *HbA1c* Glycated hemoglobin A1c, *IQR* Interquartile range, *RHD* Rapid hip destruction, *WBC* White blood cell.

*Mann–Whitney’s U test.

^||^Welch’s T-test.

^§^Statistically significant.

### Combination of inflammatory and nutritional markers

The albumin/globulin ratio (AGR), CRP-albumin ratio (CAR), Glasgow prognostic score (GPS), modified Glasgow prognostic score (mGPS), prognostic nutrition index (PNI), Geriatric Nutritional Risk Index (GNRI), neutrophil/lymphocyte ratio (NLR), platelet/lymphocyte ratio (PLR), and lymphocyte/monocyte ratio (LMR) were calculated from the serum inflammatory and nutritional marker (Table 3). The CAR, GPS, mGPS, and PLR were significantly higher, while the AGR, PNI, and GNRI were significantly lower in the infectious RHD patients, although the NLR and LMR were not significantly different between infectious and non-infectious RHD patients (Table 3).

**Table 3:** Patient laboratory, inflammation and nutrition data.

Inflammation and nutrition data	All patients (n = 50)	Patients with infectious RHD (n = 13)	Patients with non-infectious RHD (n = 37)	*p* value
AGR (IQR)	1.4 (1.0–1.5)	0.8 (0.7–1.0)	1.4 (1.3–1.6)	< 0.001*^§^
CAR (IQR)	1.72 (0.26–5.74)	18.2 (13.4–34.6)	0.68 (0.16–2.00)	< 0.001*^§^
GPS, points (IQR)	0 (0–2)	2 (1–2)	0 (0–0)	< 0.001*^§^
mGPS, points (IQR)	1 (0–2)	2 (1–2)	0 (0–1)	< 0.001*^§^
PNI (IQR)	45.4 (40.4–49.0)	37.1 (33.7–44.4)	48.2 (44.0–49.3)	< 0.001*^§^
GNRI (IQR)	102.6 (96.1–107.1)	91.0 (82.8–101.4)	104.1 (101.3–110.3)	0.001*^§^
NLR (IQR)	3.80 (2.71–5.54)	4.46 (3.44–10.8)	3.80 (2.44–5.31)	0.199*
PLR (IQR)	23.0 (15.7–32.7)	34.7 (26.8–50.6)	18.7 (13.7–29.3)	0.006*^§^
LMR (IQR)	2.70 (2.05–3.82)	2.23 (1.84–3.52)	2.72 (2.22–3.89)	0.303*

*AGR* Albumin/globulin ratio, *CAR* CRP-albumin ratio, *GNRI* Geriatric nutritional risk index, *GPS* Glasgow prognostic score, *IQR* Interquartile range, *LMR* Lymphocyte/monocyte ratio, *mGPS* Modified Glasgow prognostic score, *NLR* Neutrophil/lymphocyte ratio, *PLR* Platelet/lymphocyte ratio, *PNI* Prognostic nutrition index, *RHD* Rapid hip destruction.

*Mann–Whitney’s U test.

^§^Statistically significant.

### Radiographical data

X-ray images showed no significant differences in the degree of femoral head destruction or presence of acetabular destruction between infectious and non-infectious RHD patients (Table 4). In MRI, bone marrow edematous change was a common finding in both groups of patients. Bone marrow edema on the acetabular side was more prevalent in infectious RHD patients than in non-infectious RHD patients (84.6% vs 59.5%, *p* = 0.173), and inflammatory changes in muscles and soft tissues surrounding the hip joint in infectious RHD patients were also more prevalent in infectious RHD patients than in non-infectious RHD patients (76.9% vs 24.3%, *p* = 0.002). Abscess formation was observed in 2 of 13 infectious RHD patients (15.4%) but was not seen in any non-infectious RHD patients (Table 4).

**Table 4:** Patient radiographical data.

Radiographical data	All patients (n = 50)	Patients with infectious RHD (n = 13)	Patients with non-infectious RHD (n = 37)	*p* value
**X-ray images**				
Degree of femoral head destruction, % (IQR)	33 (20–47)	39 (17–58)	31 (21–47)	0.799*
Acetabular destruction, n (%)	25 (50.0)	7 (53.8)	18 (48.6)	1.000^†^
**MRI images**				
Bone marrow edema of the femoral head, n (%)	48 (96.0)	13 (100)	35 (94.6)	1.000^†^
Bone marrow edema of the acetabulum, n (%)	33 (66.0)	11 (84.6)	22 (59.5)	0.173^†^
Abscess, n (%)	2 (4.0)	2 (15.4)	0 (0)	0.064^†^
High signals of the hip joint surrounding muscles, n (%)	19 (38.0)	10 (76.9)	9 (24.3)	0.002^†§^

*IQR* Interquartile range, *RHD* Rapid hip destruction.

*Mann–Whitney’s U test.

^†^Fisher’s exact test.

^§^Statistically significant.

### Microbiological test

#### Preoperative joint aspiration culture (PAC)

Among the 50 patients, 16 (1, infectious RHD; 15, non-infectious RHD; *p* = 0.00391) did not undergo PAC due to a dry tap. PAC was positive in 10 patients with infectious RHD and was negative in all non-infectious RHD patients (*p* < 0.001) (Table 5).

**Table 5:** Patients tissue microbiological culture and histopathology data.

Tissue microbiological culture and histopathology data	All patients (n = 50)	Patients with infectious RHD (n = 13)	Patients with non-infectious RHD (n = 37)	*p* value
**PAC**				
Dry tap, n (%)	16 (32.0)	1 (7.7)	15 (40.5)	0.0391^†§^
Culture positive, n (%)	10 (20.0)	10 (76.9)	0 (0)	< 0.001^†§^
**Surgical specimen tests**				
Culture positive, n (%)	3 (6.0)	3 (23.1)	0 (0)	0.0146^†§^
Neutrophilic infiltration > 5/HPF, n (%)	14 (28.0)	10 (76.9)	4 (10.1)	< 0.001^†§^
Neutrophilic infiltration > 10/HPF, n (%)	7 (14.0)	7 (61.5)	0 (0)	< 0.001^†§^

*HPF* High-powered field, *IQR* Interquartile range, *PAC* Preoperative joint aspiration culture, *RHD* Rapid hip destruction.

^†^Fisher’s exact test.

^§^Statistically significant.

#### Tissue microbiological culture and pathology

All cultures for surgical tissue were negative in non-infectious RHD patients (Table 5). Microbiological cultures for the surgical tissue were positive in only 3 patients, all of whom were infectious RHD patients. However, there were false negatives in 10 patients with infectious RHD. Out of the 10 false-negative results, 7 had pre-administration of antibiotics (Supplementary Table S3). In the histopathologic assessment, a slight neutrophilic infiltrate (5 cells/HPF) was observed in 10 cases of infectious RHD and in 4 cases of non-infectious RHD patients; definitive neutrophilic infiltration (10 cells/HPF) was observed in 7 of the infectious RHD patients (Table 5).

#### Diagnostic accuracy of preoperative infectious RHD examinations

We calculated the diagnostic accuracies of imaging and serum examinations for infectious RHD (Tables 6, 7). CRP and CAR had the largest area under the curve (AUC) at 0.99, followed by GPS and AGR at 0.97 and 0.96, respectively (Table 7 and Supplementary Fig. S1). In terms of test accuracy, the sensitivity was 1.00 for the CRP, albumin level, CAR, GPS, and PNI and the specificity was 0.95 for the AGR and mGPS, 0.94 for the PLR, and 0.92 for the CAR. Among the imaging and serum examinations, the serum inflammatory marker CRP (cut-off value of 10 mg/L) and nutrition marker Albumin (cut-off value of 4.0 g/dL) showed the highest sensitivity for the diagnosis of infectious RHD (1.00 for both; specificity of 0.87 and 0.73, respectively), indicating that they were useful for screening infection in RHD patient. Then, CAR in combination with CRP and Albumin showed a sensitivity of 1.00 and specificity of 0.92, improving the diagnostic accuracy over each alone. In addition, an assessment of nutrition in combination with the inflammatory marker AGR and mGPS showed the highest specificity for infectious RHD (0.95 for both; sensitivity of 0.92 and 0.69, respectively), indicating the supplemental effect of nutritional evaluation for RHD patients (Table 7).

**Table 6:** Diagnostic accuracy of the tests of infection.

Index	Accuracy (95% CI)	Sensitivity (95% CI)	Specificity (95% CI)	PPV (95% CI)	NPV (95% CI)
MRI: inflammatory spread to soft tissues surrounding hip joints	0.76 (0.62–0.87)	0.77 (0.46–0.95)	0.76 (0.59–0.88)	0.53 (0.29–0.76)	0.90 (0.74–0.98)
PAC: positive	0.94 (0.84–0.99)	0.77 (0.46–0.95)	1.00 (0.86–1.00)	1.00 (0.59–1.00)	0.93 (0.80–0.98)
Neutrophilic infiltration > 5/HPF	0.86 (0.73–0.94)	0.77 (0.46–0.95)	0.89 (0.75–0.97)	0.71 (0.42–0.92)	0.92 (0.78–0.98)
Neutrophilic infiltration > 10/HPF	0.88 (0.76–0.96)	0.54 (0.25–0.81)	1.00 (0.86–1.00)	1.00 (0.47–1.00)	0.86 (0.72–0.95)

*CI* Confidence interval, *MRI* Magnetic resonance imaging, *NPV* Negative predictive value, *PAC* Preoperative joint aspiration culture, *PPV* Positive predictive value, *WBC* White blood cell.

**Table 7:** Diagnostic accuracy of the markers of infection.

Index	Cut-off	AUC (95% CI)	Sensitivity (95% CI)	Specificity (95% CI)	PPV (95% CI)	NPV (95% CI)
CRP, mg/L	> 10	0.99 (0.97–1.00)	1.00 (0.66–1.00)	0.87 (0.71–0.96)	0.72 (0.47–0.90)	1.00 (0.84–1.00)
ESR, mm/h	> 30	0.95 (0.90–1.00)	0.85 (0.55–0.98)	0.84 (0.68–0.94)	0.65 (0.38–0.86)	0.94 (0.80–0.99)
WBC count, 10*3/μL	> 7.3	0.69 (0.53–0.86)	0.69 (0.39–0.91)	0.68 (0.50–0.82)	0.43 (0.22–0.66)	0.86 (0.68–0.96)
Platelet count, 10*4/μL	> 3.3	0.86 (0.73–0.99)	0.77 (0.46–0.95)	0.84 (0.68–0.94)	0.63 (0.35–0.85)	0.91 (0.76–0.98)
Albumin, g/dL	< 4.0	0.93 (0.86–1.00)	1.00 (0.66–1.00)	0.73 (0.56–0.86)	0.57 (0.35–0.77)	1.00 (0.82–1.00)
AGR	< 1.1	0.96 (0.91–1.00)	0.92 (0.64–1.00)	0.95 (0.82–0.99)	0.86 (0.57–0.98)	0.97 (0.86–1.00)
CAR	> 4.56	0.99 (0.97–1.00)	1.00 (0.66–1.00)	0.92 (0.78–0.98)	0.81 (0.54–0.96)	1.00 (0.85–1.00)
GPS, points	> 0	0.97 (0.94–1.00)	1.00 (0.66–1.00)	0.81 (0.65–0.92)	0.65 (0.41–0.85)	1.00 (0.83–1.00)
mGPS, points	> 1	0.92 (0.85–0.99)	0.69 (0.39–0.91)	0.95 (0.82–0.99)	0.82 (0.48–0.98)	0.90 (0.76–0.96)
PNI	< 46.4	0.87 (0.77–0.97)	1.00 (0.66–1.00)	0.62 (0.44–0.78)	0.50 (0.30–0.70)	1.00 (0.77–1.00)
GNRI	< 98	0.81 (0.65–0.97)	0.69 (0.39–0.91)	0.81 (0.65–0.92)	0.56 (0.30–0.80)	0.88 (0.73–0.97
PLR	> 34.7	0.76 (0.58–0.94)	0.54 (0.25–0.81))	0.94 (0.80–0.99)	0.78 (0.40–0.97)	0.84 (0.69–0.94)

*AGR* Albumin/globulin ratio, *AUC* Area under the curve, *CAR* CRP-albumin ratio, *CI* Confidence interval, *CRP* C-reactive protein, *ESR* Erythrocyte sedimentation rate, *GNRI* Geriatric nutritional risk index, *GPS* Glasgow prognostic score, *mGPS* Modified Glasgow prognostic score, *NPV* Negative predictive value, *PLR* Platelet/lymphocyte ratio, *PNI* Prognostic nutrition index, *PPV* Positive predictive value, *WBC* White blood cell.

## Discussion

Preoperative diagnosis of infection is important for optimal treatment selection for RHD patients. As this study aimed to investigate the characteristics of imaging or serum markers in infectious and non-infectious RHD and to assess the data’s preoperative diagnostic accuracy to differentiate between the two RHDs, we first evaluated patients’ demographic and medical data. Although diabetes mellitus, chronic renal failure with hemodialysis, and immunosuppressive drug use have been reported as risks for SAH, which may be a major cause of infectious RHD^21,22^, these comorbidities were not associated with the presence of infection.

We found significant differences in serum inflammatory and nutritional markers between the infectious and non-infectious RHD patients. As previously reported, testing with a serum CRP cut-off value of 10 mg/L was a useful and simple screening method for infection even with RHD^14,23^. Although low specificity has been noted as a limitation of using CRP levels for diagnosis of orthopedic infections, our study’s specificity was high, perhaps because we only included severely destructive infection cases. In hematologic analysis, platelet and WBC counts were determined as other useful markers for infection detection. The relationship between platelets and infections is complex, and not all underlying mechanisms have been fully elucidated. However, platelets may be involved in innate immunity and can be activated during bacterial infections, which could explain our observed increase of platelets^24^.

A novel finding of this study is that serum nutritional evaluation, especially the albumin level, can be useful for the preoperative infectious RHD diagnosis. Albumin is a widely accepted clinical indicator for systemic nutritional status; it is occasionally utilized as an indicator of inflammation or infection because continuously increased albumin consumption during inflammation or infection can result in decreased albumin synthesis and lower serum levels^25^. Association of hypoalbuminemia with increased risk of surgical site infection in orthopedic surgeries^26^ supports our finding that the albumin level decreases in association with RHD infection. In addition, increased immunoglobulin production during infection results in a dissociation between total protein and albumin. Therefore, we focused on the combination indexes of inflammation and nutrition. For instance, the AGR was markedly decreased in the infectious RHD patients, and the AGR, CAR, mGPS, and PLR were determined as specific indices for the differentiation between infectious and non-infectious RHD (Table 7). The importance of nutritional assessment has been demonstrated in oncology^15^ and is increasingly reported in orthopedics patients^16–19^. However, this study may be the first to report the utility of these combination indexes in RHD infection diagnosis. The evaluation of serum nutritional markers may be useful for infectious RHD diagnosis, especially in patients using pre-administration of antibiotics that conceal infectious inflammation and reduce the diagnostic accuracy of inflammatory markers.

Image diagnosis for RHD patients is complex, because femoral head destruction itself can cause inflammation in bones and soft tissue. In this study, plain X-rays were demonstrated as not useful for diagnosing infection^27^. Basic MRI is useful for evaluating bone marrow edema, femoral head necrosis, synovial effusion, and inflammatory changes around the hip^28,29^, but these were not effective enough to accurately identify the infection here. MRI provides vital information about the inflammatory area, but this may not be explicitly useful for diagnosing infectious RHD, with the exception of identifying abscess formation.

Detection of causative bacteria by PAC or tissue culture is the gold standard for preoperative diagnosis of SAH^21^. However, our study indicated that low sensitivity is one of microbiological culture’s major drawbacks, despite its high specificity. PAC can provide definitive diagnostic information, but the reliability is much lower than with tissue culture, due to a dry tap, skin contamination, or false-negative results due to valuable but noncultuerable (VBNC)^11,12^. Preoperative antimicrobial use was suspected as one of the reasons for the VBNC status of bacteria^30^, and the addition of histopathologic assessments can improve the diagnostic accuracy of tissue examination, as shown in periprosthetic joint infection diagnosis^31^.

One of the limitations of this retrospective observational study is that the diagnosis for infectious RHD was based on modified diagnostic criteria in PJI, since the pathogenesis of the RHD condition remains unclear. There are classical SAH diagnostic methods using clinical and other findings, but in the case of RHD patients, these methods are inadequate to differentiate the infection condition. Therefore, the high accuracy with CRP may be because it was included in the diagnostic criteria here. However, evaluation of serum albumin was demonstrated to improve the accuracy of CRP for infectious RHD, indicating the potential importance of nutritional condition in differentiation of pathogenesis of RHD. Another limitation here is the small number of patients in our investigation, RHD being a relatively rare condition. Studies with a larger number of hip disorder patients will determine whether serum nutritional assessment is useful in SAH diagnosis. Finally, this study used general nutritional assessment parameters to assess the effect of nutritional status in an infection diagnosis. However, these scores were not developed for diagnosing infection and need to be validated in orthopedic disorders to develop a scoring system that combines multiple items in orthopedic infection diagnosis^32^.

## Conclusion

Evaluation of inflammatory markers (CRP, ESR, and platelets) is a sensitive screening method, and evaluation of nutritional and immune status (AGR, CAR, mGPS, and PLR) can improve the diagnostic reliability of inflammatory markers to differentiate between infectious and non-infectious RHD.

## Methods

This single-center, retrospective observational study investigation complied with the Declaration of Helsinki and was approved by the Ethics Committee of Yokohama City University Hospital (approval no: B200200007). Informed consent was obtained from all the study participant by opt-out method on the web-site. Sixty-seven patients who underwent THA between April 2008 and April 2020 due to destructive changes in femoral heads within 1 year from the clinical onset were investigated. Cases were excluded if the femoral head was not submitted for pathological assessment. As a result, 50 cases who had complete demographic, serological, radiographical, histological, and pathological data were enrolled in this study (Fig. 1).

### Demographic data

Patient demographic data included age, sex, diagnosis, height, weight, BMI, functional HHS, time until femoral head destruction, time from onset to surgery, diabetes mellitus, hemodialysis, steroid use, and antimicrobial use at the time of the first surgery (Table 1).

### Clinical, laboratory, and nutritional assessment

Serological data analysis included the collection of data on the following: HbA1c, WBC count (10^3^/μL), total lymphocyte and neutrophil count (10^3^/μL), monocyte count (10^2^/μL), platelet count (10^4^/μL), total protein (g/dL), albumin (g/dL), CRP (mg/L), and ESR (mm/h) (Table 2). We calculated the AGR, CAR, GPS, mGPS, PNI, GNRI, NLR, PLR, and LMR. These metrics indicate systemic nutritional status, systemic inflammation, and immune status (Supplementary Table S2)^16,17,22,33–42^. All data were calculated from blood samples obtained during the first visit to our hospital after the appearance of clinical onset with femoral destruction.

### Radiographical analysis

Plain hip joint X-rays and MRIs at the first hospital visit after femoral destruction were utilized for the radiographic analysis. The degree of femoral head destruction and presence of acetabular destruction were assessed on X-ray images. Femoral head destruction was calculated by comparing the ratio of the diameter of the residual femoral head to that of the original femoral head, approximated by drawing a perfect circle on the residual bone. Erosion, irregularity, and obvious destruction of subchondral bone on the acetabular side were defined as acetabular destruction. Bone marrow edema or inflammatory changes were assessed in the acetabulum and proximal femur, using MRI with T1- and T2-weighted and fat-suppressed images. Abscess formation or inflammatory changes in muscles and soft tissues surrounding the hip joint were also assessed on fat-suppressed MRI.

### Microbiological testing

#### Preoperative joint aspiration culture (PAC)

PAC was conducted in all patients. All samples were sent to the microbiology laboratory. First, all samples underwent standard culture using 5% sheep’s blood agar plates containing peptone and sodium chloride. Standard culture was performed for 24 h at 35 °C/5% CO_2_. If a sample was negative in a standard culture, the sample underwent a 5-day enrichment culture procedure. The enrichment culture used a semi-solid Gifu anaerobic medium containing peptone, soy peptone, protease peptone, beef extract, yeast extract, liver extract, glucose, starch, l-tryptophan, l-cysteine hydrochloride, thioglycolic acid sodium salt, l-arginine, vitamin K1, hemin, potassium dihydrogen phosphate, and sodium chloride. The enrichment culture was performed for 5 days at 35 °C in ambient air composition. When a sample was negative in the enrichment culture, it was culture-negative^43^. Bacteria were identified through analysis of biological properties using MicroScan WalkAway system with combo panels (Beckman Coulter, Brea, CA).

#### Tissue microbiological culture and pathology

Surgical tissues were postoperatively assessed by microbiological culture and histopathological examination. The bacterial culture test of surgical tissues was performed in the same way as the PAC, with a 24-h standard culture, and if negative, followed by a 5-day incremental culture for determination. The femoral head was processed for histopathological assessment after dissection, decalcification, and hematoxylin and eosin staining. Neutrophil infiltration was considered as a finding for infectious RHD when five or more neutrophils were found in a single high-powered field (HPF) under the microscope^32,44^. Other specific histopathologic findings suggestive of bone necrosis, rheumatoid arthritis, and a benign or malignant tumor were noted.

#### Definition of the diagnosis of infectious or non-infectious diseases

Infectious RHD was diagnosed when more than two of the following tests were positive: 1. CRP > 10 mg/L or ESR > 30 mm/h, 2. Detection of a causative pathogen in the microbiological culture, and 3. Positive for infection in histopathological assessment. In non-infectious RHD, ONFH was diagnosed using the diagnostic criteria for ONFH^45^, and subchondral fracture was diagnosed if the histopathology findings excluded other specific conditions, including tumors or rheumatoid arthritis (Fig. 2).

### Statistical analysis

All statistical analyses were performed with EZR (Saitama Medical Center, Jichi Medical University, Saitama, Japan, version 1.40)^46^, which is a graphical user interface for R (The R Foundation for Statistical Computing, Vienna, Austria, version 3.5.2). For continuous variables that were not equally distributed, we listed the median and provided the interquartile range. Student’s t-tests were used for two-group comparisons when continuous variables were normally distributed and the data were equally distributed. Welch’s t-tests were performed when the data were not equally distributed. Mann–Whitney U tests were used for continuous variables that were not normally distributed, and Fisher exact tests were employed for categorical variables. Sensitivity, specificity, positive and negative predictive values, and accuracy for the diagnosis of infectious RHD were calculated based on the definitive diagnosis. All statistical tests were two-sided, and p-values < 0.05 were considered statistically significant.

## Supplementary Information

Supplementary Information.

## Data Availability

The data presented in this study are available on request from the corresponding author. The data are not publicly available due to conditions of the ethics committee of our university.

## Abbreviations

AGR: Albumin/globulin ratio
AUC: Area under the curve
BMI: Body mass index
CAR: CRP-albumin ratio
CI: Confidence interval
CRP: C-reactive protein
ESR: Erythrocyte sedimentation rate
GNRI: Geriatric nutritional risk index
GPS: Glasgow prognostic score
HbA1c: Glycated hemoglobin A1c
HPF: High-powered field
IQR: Interquartile range
LMR: Lymphocyte/monocyte ratio
mGPS: Modified Glasgow prognostic score
MRI: Magnetic resonance imaging
MSIS: Musculoskeletal Infection Society
NLR: Neutrophil/lymphocyte ratio
NPV: Negative predictive value
ONFH: Osteonecrosis of the femoral head
PAC: Preoperative joint aspiration culture
PJI: Periprosthetic joint infection
PLR: Platelet/lymphocyte ratio
PNI: Prognostic nutrition index
PPV: Positive predictive value
RDC: Rapidly destructive coxarthrosis
RHD: Rapid hip destruction
SAH: Septic arthritis of the hip
THA: Total hip arthroplasty
VBNC: Valuable but noncultuerable
WBC: White blood cell
