# Supplementary material for: Inflammation and nutrition based screening tests for detection of infection in cases of rapid hip destruction
Source: Sci Rep. 2022 Mar 4;12:3586. doi: 10.1038/s41598-022-07678-3 (PMC8897466; doi:10.1038/s41598-022-07678-3)
Supplement: Supplementary file 1 — Supplementary Information. [file 41598_2022_7678_MOESM1_ESM.docx]

**Inflammation and Nutrition based Screening tests for**

**Detection of Infection**

**in Cases of Rapid Hip Destruction**

Koki Abe^1^, MD, Hyonmin Choe^1^, MD, PhD*,

Masatoshi Oba^1^, MD, PhD, Taro Tezuka^1^, MD, PhD, Hiroyuki Ike^1^, MD, PhD,

Naomi Kobayashi^2^, MD, PhD, Yutaka Inaba^1^, MD, PhD

^1^Yokohama City University, Department of Orthopaedic Surgery

^2^ Yokohama City University Medical Center, Department of Orthopaedic Surgery

**Running title**: Nutritional evaluation for RHD

*Correspondence to:

Hyonmin Choe, MD, PhD

Department of Orthopaedic Surgery, Yokohama City University

3-9 Fukuura, Kanazawa-Ku, Yokohama 236-0004, Japan

Phone: +81-45-787-2655, Fax: +81-45-781-7922

Email: [hyonmin@hotmail.com](mailto:hyonmin@hotmail.com)

**Supplementary Tables**

Supplementary Table S1. Demographic data for non-infectious RHD patients

| **Demographic data** | **All patients**  **(n = 37)** | **Patients with ONFH**  **(n = 13)** | **Patients with RDC**  **(n = 24)** | **p-value** |
| --- | --- | --- | --- | --- |
| Patient age, yrs (IQR) | 65 (57-75) | 57 (18-75) | 68 (43-89) | 0.003^*§^ |
| Female patients, n (%) | 23 (62) | 5 (39) | 18 (75) | 0.039^†§^ |
| BMI, kg/m^2^ (IQR) | 22.8 (20.3-25.6) | 21.3 (18.8-28.5) | 23.4 (17.8-37.6) | 0.115^*^ |
| Time until femoral head destruction, months (IQR) | 4 (2.75-6) | 5.5 (3-6) | 3 (1-11) | 0.172^*^ |
| Time from the first onset to surgery,  months (IQR) | 6.5 (3-12) | 8 (3-22) | 5 (1-18) | 0.109^*^ |
| Harris hip score, points (IQR) | 44 (30-54) | 52 (23-68) | 42 (20-61) | 0.108^*^ |
| Diabetes mellitus, n (%) | 19 (51) | 9 (69) | 10 (42) | 0.170^†^ |
| Hemodialysis, n (%) | 4 (10.8) | 2 (15.4) | 2 (8.3) | 0.602^†^ |
| Use of steroids, n (%) | 12 (32) | 8 (62) | 4 (17) | 0.010 ^†§^ |
| Use of antibiotics, n (%) | 4 (11) | 1 (8) | 3 (13) | 1.000^†^ |

BMI: body mass index; IQR: interquartile range; ONFH: osteonecrosis of the femoral head; RDC: rapidly destructive coxarthrosis; RHD: rapid hip destruction

* Mann-Whitney’s U test; † Fisher’s exact test; § statistically significant

Supplementary Table S2. Nutrition evaluation

| **Index** | **Description** | | **Previous report** |
| --- | --- | --- | --- |
| Albumin | An important indicator of nutrition that accounts for most of the total protein in the blood; hypoalbuminemia is a risk for SSI^[26]^ | | <3.5 g/dL: SSI risk^[26]^ |
| Albumin/globulin ratio (AGR) | Comprehensive markers of nutrition and immunity focusing on changes in protein composition. Low AGR is indicative of PJI^[42]^ | | <1.2: PJI suspected^[42]^ |
| CRP-albumin ratio (CAR) | Calculated by dividing CRP (mg/L) by albumin (g/dL)  High values indicating a worse prognosis for infection^[40]^ and poor prognosis for hip fracture^[35]^ | | >2.49: a strong indicator for 1-year mortality in patients operated on due to hip fracture^[35]^ |
| Glasgow prognostic score  (GPS) | The state of systemic inflammation, reported in a variety of cancers, was assessed by combining CRP and albumin | | > 0: HR 1.95, poorer overall survival in osteosarcoma patients^[39]^ |
|  | CRP ≦10 mg/L and Alb ≧3.5 g/dL  CRP >10 mg/L or Alb <3.5 g/dL  CRP >10 mg/L and Alb <3.5 g/dL | 0 point  1 point  2 point |  |
| Modified Glasgow prognostic score  (mGPS) | The state of systemic inflammation; the modified GPS is more a CRP-weighted score than the original GPS^[41]^ | | Higher points predicted a higher risk of postoperative mortality in stage II and/or III colorectal cancer patients^[41]^ |
|  | CRP ≦5 mg/L and Alb ≧3.5 g/dL  CRP >5 mg/L or Alb <3.5 g/dL  CRP >5 mg/L and Alb <3.5 g/dL | 0 point  1 point  2 point |  |
| Prognostic nutrition index  (PNI) | Immunocompetence and nutritional status  10*Alb＋0.005×Total lymphocyte count | | <47: an independent predictor of postoperative morbidity in gastric cancer patients |
| Geriatric Nutritional Risk Index  (GNRI) | Used to evaluate nutritional risk index; in patients with hip fracture, it was related to the 100-m walking speed^[38]^  1.489*albumin(g/L)+(41.7*weight(kg)/ideal body weight)^[32]^ | | >98: no risk^[36]^  92-98: mild risk  <92: major risk |
| Neutrophil/lymphocyte ratio  (NLR) | Indicators of inflammation were noted as a marker of infection and inflammation^[19]^ | | >2.77: PJI is suspected^[19]^  (sensitivity: 0.846, specificity: 0.897) |
| Platelet/lymphocyte ratio  (PLR) | Indicators of inflammation were noted as a marker of infection and inflammation^[19]^ | | >139.22: PJI is suspected^[19]^  (sensitivity: 0.885, specificity: 0.872) |
| Lymphocyte/monocyte ratio  (LMR) | Indicators of inflammation were noted as a marker of infection and inflammation^[19]^ | | <3.40: PJI is suspected^[19]^  (sensitivity: 0.885, specificity: 0.782) |

CRP: C-reactive protein; HR: hazard ratio; PJI: periprosthetic joint infection; SSI: surgical site infection

Supplementary Table S3. Diagnosis of infectious RHD

| **No.** | **Antibiotic**  **use** | **CRP** | **ESR** | **Abscess** | **Culture** | **Neutrophilic infiltration** |
| --- | --- | --- | --- | --- | --- | --- |
| 1 | + | 6.55 | 111 | - | *Staphylococcus aureus*^¶^ | ++ |
| 2 | + | 29.94 | 100 | - | *Staphylococcus aureus*^¶^ | - |
| 3 | - | 15.93 | 103 | - | *Staphylococcus aureus*^¶^ | ++ |
| 4 | - | 4.82 | 76 | - | *Klebsiella pneumoniae* (ESBL)^⊥^ | ++ |
| 5 | + | 28.3 | 111 | + | *Streptococcus agalactie*^¶^ | - |
| 6 | + | 5.64 | 87 | - | *Staphylococcus epidermidis* *^¶^ | ++ |
| 7 | - | 1.46 | 61 | - | *Staphylococcus aureus*^¶^  *Staphylococcus warneri*, *Staphylococcus species*^⊥^ | + |
| 8 | + | 1.94 | 35 | - | - | + |
| 9 | + | 3.17 | 20 | - | *Staphylococcus haemolyticus*^¶^  *Staphylococcus epidermidis** ^⊥^ | ++ |
| 10 | - | 5.4 | 74 | - | *Staphylococcus aureus**^¶^ | ++ |
| 11 | - | 4.96 | 58 | - | *Staphylococcus aureus**^¶^ | - |
| 12 | + | 9.69 | 89 | - | - | ++ |
| 13 | + | 9.61 | 27 | + | *Staphylococcus aureus*^¶^ | + |

CRP: C-reactive protein; ESR: erythrocyte sedimentation rate; RHD: rapid hip destruction

* methicillin-resistant strain; ¶ Preoperative joint fluid culture result; ⊥ Surgical specimen culture result.

+ Neutrophilic infiltration >5/HPF; ++ Neutrophilic infiltration >10/HPF.

**Supplementary Figure**





Supplementary Fig. S1. Receiver operating characteristic curves of the serological tests.

AGR: albumin/globulin ratio; CAR: CRP-albumin ratio; CRP: C-reactive protein; ESR: erythrocyte sedimentation rate; GNRI: Geriatric Nutritional Risk Index; GPS: Glasgow prognostic score; mGPS: modified Glasgow prognostic score; PLR: platelet/lymphocyte ratio; PNI: prognostic nutrition index; WBC: white blood cell
